# Application of Correlation Analysis for Assessment of Infrasound Signals Emission by Wind Turbines

**DOI:** 10.3390/s20236891

**Published:** 2020-12-02

**Authors:** Tomasz Boczar, Dariusz Zmarzły, Michał Kozioł, Daria Wotzka

**Affiliations:** Faculty of Electrical Engineering Automatic Control and Informatics, Opole University of Technology, 45-758 Opole, Poland; t.boczar@po.edu.pl (T.B.); d.zmarzly@po.edu.pl (D.Z.); d.wotzka@po.edu.pl (D.W.)

**Keywords:** infrasound measurement system, wind turbine, infrasound correlation analysis

## Abstract

The study reported in this paper is concerned with areas related to developing methods of measuring, processing and analyzing infrasound noise caused by operation of wind farms. The paper contains the results of the correlation analysis of infrasound signals generated by a wind turbine with a rated capacity of 2 MW recorded by three independent measurement setups comprising identical components and characterized by the same technical parameters. The measurements of infrasound signals utilized a dedicated measurement system called INFRA, which was developed and built by KFB ACOUSTICS Sp. z o.o. In particular, the scope of the paper includes the results of correlation analysis in the time domain, which was carried out using the autocovariance function separately for each of the three measuring setups. Moreover, the courses of the cross-correlation function were calculated separately for each of the potential combinations of infrasound range recorded by the three measuring setups. In the second stage, a correlation analysis of the recorded infrasound signals in the frequency domain was performed, using the coherence function. In the next step, infrasound signals recorded in three setups were subjected to time-frequency transformations. In this part, the waveforms of the scalograms were determined by means of continuous wavelet transform. Wavelet coherence waveforms were calculated in order to determine the level of the correlation of the obtained dependencies in the time-frequency domain. The summary contains the results derived from using correlation analysis methods in the time, frequency and time-frequency domains.

## 1. Introduction

The task of the adequate recording of infrasound signals generated by sources of emission poses a relatively difficult measurement task in practical application. This is attributable to the possibility of a number of potential sources responsible for generating acoustic signals in the low frequency bandwidth, including infrasound that accompanies normal operation of wind turbines. Such sources include noise caused by vehicle traffic, agricultural machines or passing trains, as well as natural ones caused by waves of the water surface or blowing winds that sweep obstacles on its way. We can bear in mind that most often wind turbines operate as part of wind farms, which include from several to even several thousand individual generators. Moreover, even a few wind farms can be located in a relatively small area in relation to the possible range of the emitted infrasound. Additionally, location-specific conditions occur in which a given wind farm comprises turbines with different technical design, made by various manufacturers, with different dimensions and capacities, which may affect the differentiation of the infrasound emission. There are wind farms in which turbines with various service lives also operate side by side. For instance, the study reported in the article [1] focused on the significant differences in the results of infrasound and acoustic noise measurements in the audible band, which were recorded close to wind farms, depending on the type of the supporting structure applied in a given turbine. The authors presented the results of measurements that were carried out in the vicinity of wind turbines with different supporting structures (in the form of a truss or tubular structure) and of different heights. On the basis of the results of a comparative analysis, it was found that wind turbines with tall towers built with trusses emit much lower noise levels than ones with towers with a tubular design, and this applies to both the audible bandwidth (the level is approximately 10 dB lower) and infrasound range (a few dB). Their level was on average 10 dB higher than the background noise level, both for audible and infrasound noise levels. However, the article [2] presents the results of a comparative analysis of infrasound generated by wind turbines equipped with a synchronous and asynchronous generator, and the article [3] assesses the impact of a number of metrological parameters on the results obtained in this respect.

We can also note that the length of the propagation of infrasound waves in the air ranges from 17 m to 340 m, which directly determines the actual effect of obstacles on wave propagation in an open space. Hence, any objects whose dimensions are smaller than the length of the propagating infrasound wavelengths do not pose an obstacle. This phenomenon leads to the inconsiderable damping of infrasound signals during their propagation in the air that results only from the distance between the source and the receiver. Therefore, infrasound waves have good propagation characteristics, and their interaction at the lowest frequency values is possible even over distances of tens of kilometers. Relatively poor damping, wavelength and frequency, combined with the possibility of standing waves that can be formed in field specific conditions, as well as the possible resonance phenomenon lead to objective difficulties in the unambiguous and adequate location of the source of infrasound generation. An important element is also related with the need to take into account the acoustic background during the measurements of infrasound emitted by wind turbines, the level of which, in many cases, may be close to the useful signals [4,5,6,7,8]. In particular, this applies to the conditions when wind speeds with values above 12–15 m/s occur during infrasound recording. In the case of wind farms comprising many wind turbines, there is usually objective difficulty in measuring the noise background, as it requires stalling all operating installations by the investor or the occurrence of wind less conditions when the wind speed is below the value when turbines can start (usually below 3 m/s).

Another important issue is also associated with the lack of identical, and in many countries a complete lack of normatively specified values with the levels of permissible long-term exposition to infrasound noise in the working environment. Moreover, these levels are constantly variable, and in many regions or provinces, local regulations and laws are enforced, which have been commonly developed under the pressures of local communities. On the other hand, the issues of infrasound noise occurring in the generally accessible environment are practically not subjected to legal regulations. Additionally, there is no single common reference method of measuring and analyzing infrasound signals. In this regard, the measurement methodology specified in the IEC 61400-11 standard is employed, and it was developed with the purpose of measuring acoustic signals emitted by wind turbines in the audible range. Only the regulations contained in Annex A.2 offer the potential to extend the bandwidth to infrasound range, but these provisions do not specify details of the procedure to be applied for infrasound measurements. This standard describes a procedure for determination of the acoustic power level of wind turbines for different wind speeds on the basis of registered changes in the acoustic pressure level. On the other hand, the authors of the article [9] argue that the currently applied techniques and methodology for measuring and analyzing acoustic noise tend to obscure the ratio of low-frequency impulse noise and infrasound in the generated spectrum of signals emitted by wind turbines. It has also been raised that the widespread use of a level A-weighting filter for infrasound analysis, which is normatively used to assess noise levels in the audible range, instead of a level G-weighting filter, forms an inadequate tool and constitutes an unreliable indicator of this assessment [10].

We can note that the problem of the potential adverse effects of infrasounds generated by the operation of wind turbines to the health of people living in their vicinity may result in the lack of consent of local communities to the installation of wind farms, even at relatively large distances from residential areas. As a consequence, a slowdown and in extreme situations even an inhibition of the development of onshore wind energy can follow this. People’s fears and, consequently, opposition of local communities, in extreme cases may even lead to the closure of operating wind farms by decision of local authorities.

Therefore, the task of developing adequate techniques of measuring and analyzing infrasound signals generated by wind turbines and assessing their potential effects on human health still poses a significant problem not only from the cognitive and scientific point of view. We can remark that this problem varies in degree depending on the country, and above all is largely determined by a derivative of the state of knowledge and awareness of the local community, which results mainly from the information policy conducted in this area by the state or, respectively, by local authorities on a specific local community scale.

The level of social acceptance of working wind turbines and projected new investments varies from country to country. We need to be aware that this acceptance is guided by a number of derivative unmeasurable and subjective factors. To a large extent, public opinion is formed by the publicity focusing on the potential adverse effect associated with the operation of wind turbines on a broadly understood human health.

The positions presented in various countries and the results of the research work carried out in this area were presented in articles [10,11,12,13,14,15,16,17,18,19,20,21,22,23,24,25,26,27,28,29,30,31,32,33,34,35,36,37,38].

The purpose of the research, whose representative results are reported in this paper, was to determine the scope and applicability of correlation analysis in the time, frequency and time-frequency domains in the analysis of infrasound signals generated by a wind turbine registered in a system consisting of three independent measurement setups comprising identical elements. The use of correlation analysis offers the possibility to verify whether and how infrasound waves propagating around the investigated wind turbine are the same, and if they differ and if they are dependent on the direction of the wind.

## 2. Wind Turbine under Study

The object of this investigation was a single, three-bladed V110 wind turbine manufactured by Vestas with a rated electrical capacity of 2.0 MW, which has been in service for over 6 years. The diameter of the turbine rotor is 110 m, the sweeping area is 9.503 m^2^ and the tower height is 120 m. The speed at which the start-up and electricity production is initiated is equal to 4.0 m/s. However, the nominal wind speed at which the rated power of 2.0 MW is achieved is 12 m/s. For safety reasons, automatic shutdown of the turbine occurs for the wind speeds of 21 m/s. This turbine design has been comprehensively tested by the manufacturer in terms of the generated acoustic signals in the audible range, and the maximum value of the sound intensity emitted during their operation is 107.6 dB. The turbine has a 690 V four-pole asynchronous generator.

The investigated turbine is located in central Poland, in a lowland area, over 1800 m from the nearest built-up areas, and the nearest tarmac road at a distance of around 2 km. Due to the low acoustic nuisance and the relatively large distance, this environment forms a source of a low level of acoustic background in the infrasound range.

## 3. Characteristics of Measurement System

Dedicated measurement system called INFRA was built by KFB ACOUSTICS (KFB Acoustics Sp. z o.o., Wrocław, Poland) and was subsequently applied to for the measurements of infrasound signals generated during the operation of the wind turbine. The system was designed for the purposes of the scientific project entitled “Numerical and experimental analysis of low-frequency acoustic phenomena generated during the operation of wind turbines” funded by the National Science Center. The system used enables measurements of acoustic signals and wind speed and direction simultaneously by application of three independent and identical measurement setups. Registrations are synchronized in time and measurement data are transmitted wirelessly using Wi-Fi technology. This way, the range of distances for which measurements can be performed can be feasibly increased, and the necessity to use connection cables which are troublesome in field conditions is eliminated. It is possible to record infrasound, comparative in any three directions in relation to the wind turbine under study.

The standard and common TCP/IP (Transmission Control Protocol/Internet Protocol) transmission protocol was applied for the transfer of measurement data. Depending on the type of applied measuring sensors, the INFRA system can be utilized both for recording acoustic signals in the audible range and in the low frequency bandwidth, including infrasound. The system consisted of the following functional elements: A base station, three independent and identical measurement setups, a weather station and a laptop. The software part of the system comprised dedicated INFRA v. 1.2 software, which offers archiving and pre-processing of recorded on-line measurement data, as well as provides the operator with the option of setting measurement parameters. The intuitive graphical interface enables the user to view real-time waveforms of infrasound signals recorded by the three independent setups, as well as instantaneous wind speed and direction values. The export of recorded data occurs in the *.mat file format and was followed by its further processing in the Matlab environment.

**Figure 1:**
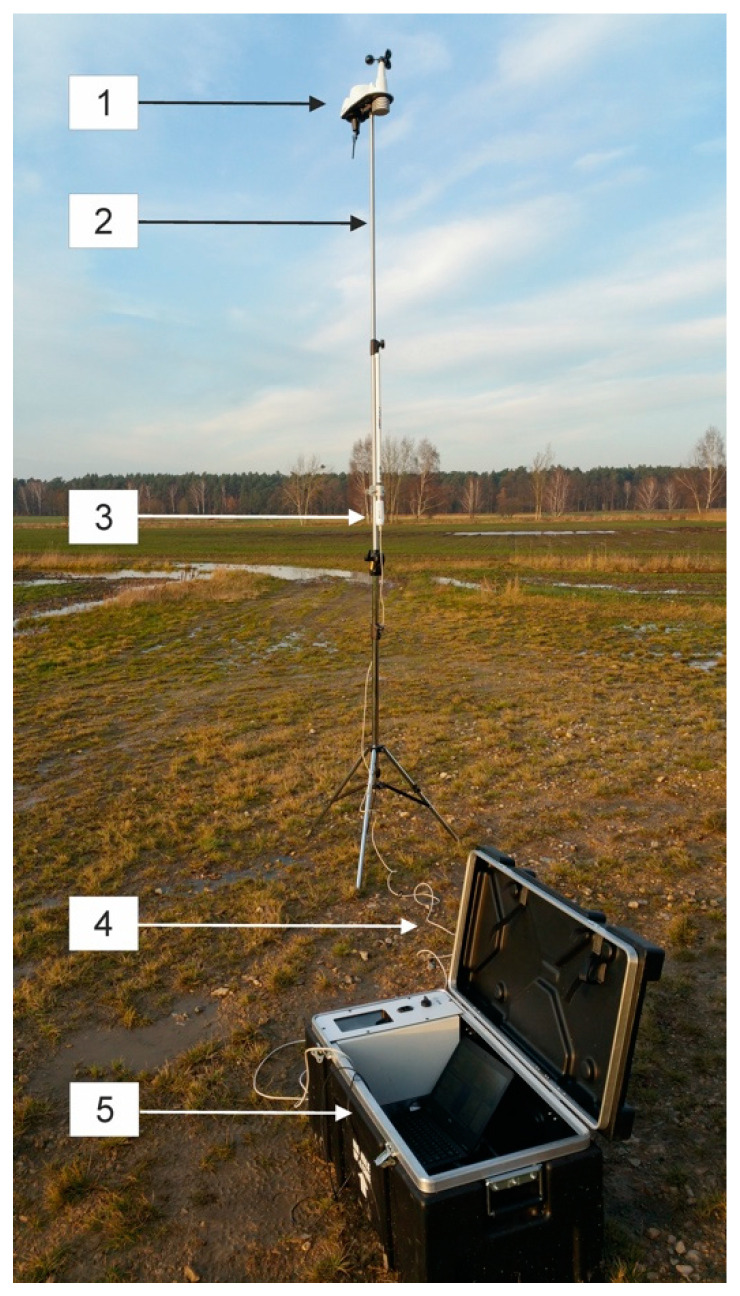
View of measuring station and weather stations, where: 1—Vantage Vue 6250EU weather station; 2—telescopic tripod; 3—ProEter104 omnidirectional antenna; 4—connector linking antenna to router and 5—measurement case with Wi-Fi 2.4 GHz access point.

The measuring station, which is presented in Figure 1, included the following functional elements: Wi-Fi router Ubiquiti BULLET M2 (Ubiquiti Networks, New York, NY, USA), omnidirectional ProEter10 CyberBajt antenna (Cyberbajt, Zamość, Poland) and a battery power supply system. Throughout the measurements, the antenna was directly coupled with the router via a dedicated connector, thus a 2.4 GHz Wi-Fi access point was created. The router comprises an Atheros MIPS 24KC, 400 MHz microprocessor and the maximum RF output power (TX Power) is 30 dBm. It is contained in a special case resistant to moisture, high temperature differences, dust and mechanical damage, i.e., to conditions that may arise during measurements performed in difficult field conditions, in which wind turbines are often installed.

With the purpose of ensuring a reliable and long-distance wireless connection with measurement stations, a vertical polarization antenna with 10 dBi energy gain was used, which was designed to operate in the 2.4 GHz bandwidth. The antenna used was dedicated to locations with a large number of other radio networks. Moreover, the large beam width in the vertical plane of 230 for −3 dB enables the implementation of connections in the case of even relatively large differences in height between the data collection system and individual setups. This can occur relatively frequently during measurements performed in field conditions. During the measurements, the antenna was placed on a 2.5 m high mast, which ensured a secure and stable connection with the antennas of measuring stations in the range of up to 300 m, with a real data transmission speed of 100 Mbps +, regardless of weather conditions and terrain type.

Additionally, in accordance with the recommendations of the PN-EN 61400-11 standard, the Davis Vantage Vue 6250EU (Davis Instruments, Hayward, CA, USA) wireless weather station was utilized for comparison purposes between infrasound signals and weather conditions accompanying measurements. During the registrations, its location was selected in accordance with the guidelines contained in the standard, i.e., in front of the turbine at a distance of 2D = 220 m (where D is the rotor diameter of the turbine). Wind speed and direction were measured both at a height of 10 m using a telescopic mast in accordance with the standard, and comparatively at a height of around 3 m using a telescopic tripod, similarly to the procedures applied for measurement setups. Its application offers air temperature measurements in the range from −40 °C to +65 °C with an accuracy of ±0.10 °C; relative humidity in the range from 0 to 100% with an accuracy of 1%; atmospheric pressure in the range from 540 hPa to 1100 hPa, with a bias of 0.1 hPa; wind speeds in the range from 1 m/s do 80 m/s (320 km/h) with an accuracy of 0.1 m/s (1 km/h) and its direction in the range from 0° to 360° with an accuracy of 10°; as well as the amount of liquid precipitation in the range from 1 to 1016 mm/h, with an accuracy of 0.2 mm. It is also possible to determine derivative parameters, such as: Dew point temperature and felt temperature. The system of external sensors, which the weather station was equipped with, communicated with the console wirelessly (frequency: 868 MHz) for a distance of up to 300 m in an open space, and following the use of additional signal amplifiers and antennas, the feasible range of distances cam increase up to 1 to 2 km. Throughout the measurements, the measurement station was connected to the DELL Latitude E7270 laptop via an Ethernet cable with the installed INFRA v. 1.2 software.

Each of the three measurement setups comprised of the following components: A measuring microphone; measurement card, battery power supply system, weather station, wind direction sensor, anemometer and external Wi-Fi antenna (Figures 3 and 4). For the measurement of infrasound emitted by wind turbines, pre-polarized a GRAS Sound & Vibration A/S free field GRAS 46AZ (Gras Sound & Vibration, Holte, Denmark) condenser microphone was employed. In this type of microphones, variations in acoustic pressure led to the diaphragm vibrations, which also forms the movable lining of the condenser. Hence, the capacitance value changes proportionally to the changes in the pressure of the acoustic wave. As the microphone is pre-polarized and its linings are connected to each other by a resistor, proportional voltage variations were obtained at the microphone output. Their main purpose was diagnostic measurements of acoustic signals in the low frequency band from 0.5 Hz to 20 kHz. These microphones meet the standards set out in the IEC 61094 WS3F standard. Moreover, they have an impulse response that is optimized for pressure measurement in a dispersed diffusion field. The values of the basic technical parameters of the microphones used are presented in Table 1.

We should note that the applied microphones were integrated via a standard BNC connector with the 26CI type preamplifiers with a gain of −0.35 dB (gain: −0.35 dB). They are characterized by a very low inherent noise level, which is typically 3.5 μV (Linear: 20 Hz−20 kHz), high dynamics and transmission bandwidth from 1 Hz to 200 kHz (±0.2 dB) (frequency range). In addition, they have a very high input impedance of 40 GΩ/0.4 pF and are effectively shielded by an annular shield to minimize the effects of dispersed capacity and direct interference from the coupled microphone.

For the purposes of effective wireless communication using Wi-Fi technology between three measuring setups and the base station, a Cyberbajt directional, microband antenna, LineEter 19 type (Cyberbajt, Zamość, Poland), with an energy gain of 19 dBi, was used for operation in the frequency bandwidth from 2.4 to 2.5 GHz. Its horizontal beam width in the horizontal plane is 250 for −3 dB and, respectively, 200 for −3 dB for the vertical plane.

The measuring station has a dedicated case made of aluminum and hardened polypropylene, which is waterproof, dustproof and impact resistant. The measuring case has been integrated with the cDAQ-9191 measurement module (National Instruments Corp., Austin, TX, USA), to which all measurement connectors have been connected and the four-channel, 24-bit NI 9234 measurement card (National Instruments Corp., Austin, TX, USA). The card used has built-in anti-aliasing filters for each of the four channels as standard, which can automatically adjust to the current sampling frequency. It is noteworthy that the maximum value of the sampling frequency is equal to 51.2 kS/s.

**Figure 2:**
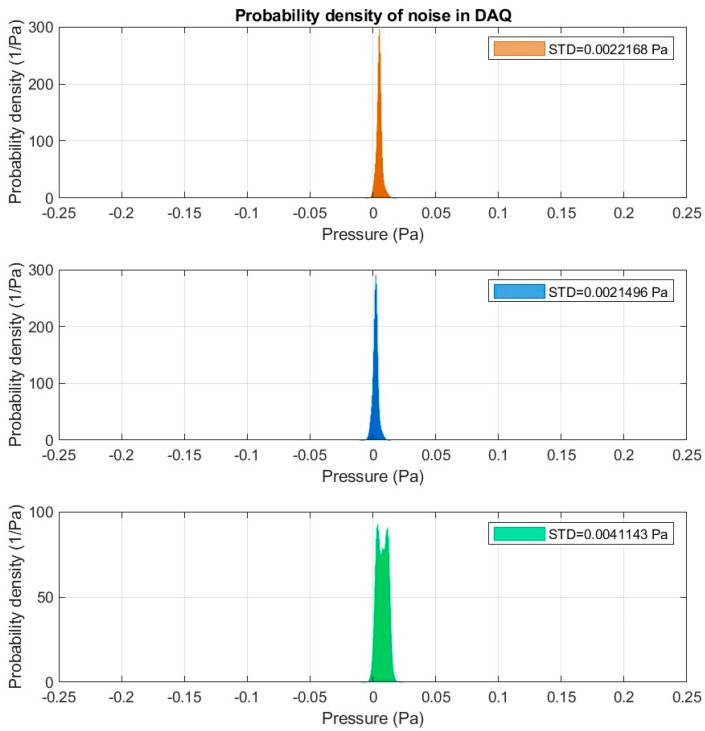
Probability density of noise in data acquisition system (DAQ): Orange color—measuring setup number 1; blue color—measuring setup number 2 and green color—measuring setup number 3.

The noise parameters of the measurement card in the idle state and the noise density for the sampling frequency were respectively: 97 dBFS (50 µV_rms_) for channel noise and 310 nV/√Hz for noise density. The self-noise density of the entire measuring system was also analyzed. The obtained results showed insignificant noise in measuring channels (Figure 2), which was taken into account when analyzing the measured signals.

**Figure 3:**
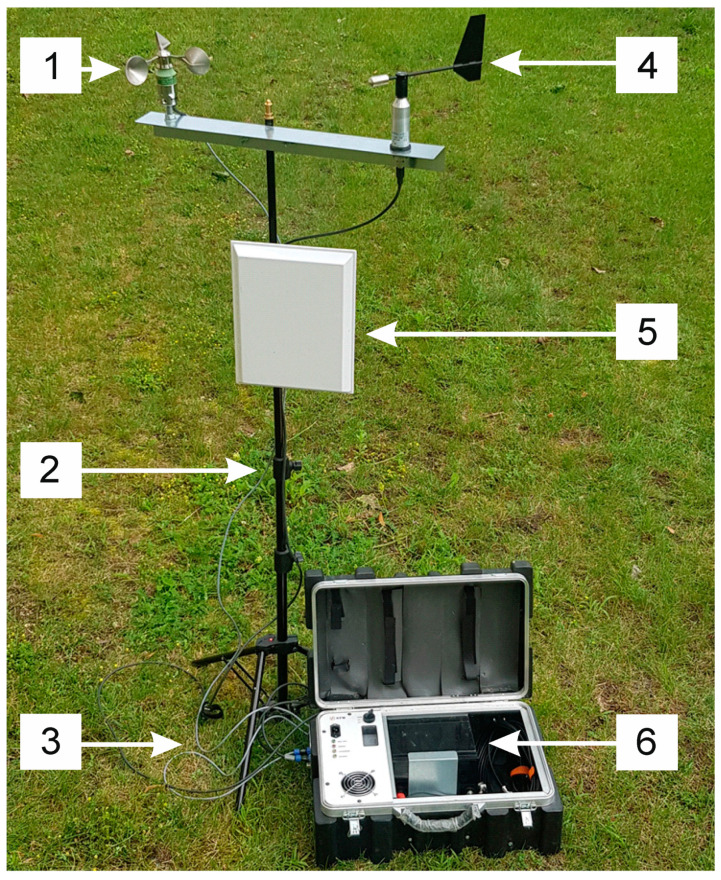
View of measuring station, where: 1—anemometer; 2—telescopic measurement tripod; 3—connector cables; 4—wind vane; 5—directional antenna and 6—measuring case.

The wind direction sensor, anemometer and an external Wi-Fi antenna were installed on a custom-made two-armed aluminum stand. The accuracy of the anemometer is ±0.1 m/s, and the wind speed measurement was in the range from 0 m/s to 25 m/s. In addition, the accuracy of the wind vane was in the range of ±10° in the range from 0° to 360° (Figure 3).

In accordance with the recommendations found in the IEC/EN 61400–11 standard that applies to measurement of audible noise generated by wind turbines, two windscreens were used for recording infrasound signals. In this way, the influence of wind gusts directly on the microphone diaphragm could be effectively eliminated, and thus their influence on the obtained results of recording was excluded.

**Figure 4:**
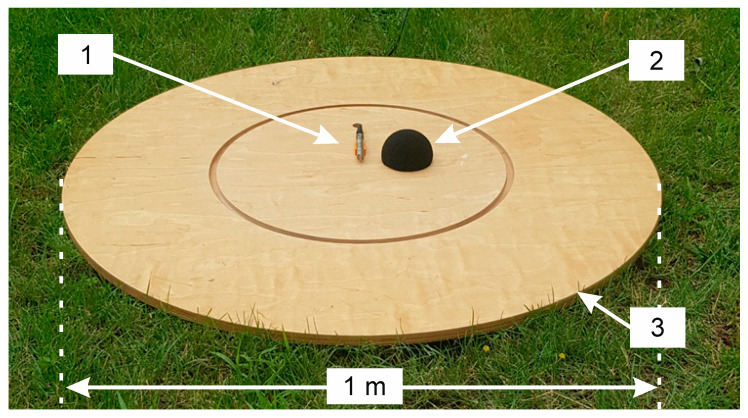
View of measuring setup, where: 1—GRAS 46AZ 26CI measuring microphone z with preamplifier located in a special holder; 2—external windscreen and 3—reflecting board.

The first one was the internal cover by Brüel & Kjær, type UA-0207 (Brüel & Kjær, Naerum, Denmark), made in the shape of a hemisphere with a radius of 4.5 cm, which was placed directly on the active part of the measuring microphone. It was made of a special polyurethane foam with open pores, which is capable to suppress noise caused by wind gusts in the range from 10 to 12 dB, depending on its speed. Its use effectively reduces the excessive air pressure exerted by wind on the measuring microphone (Figure 4).

**Figure 5:**
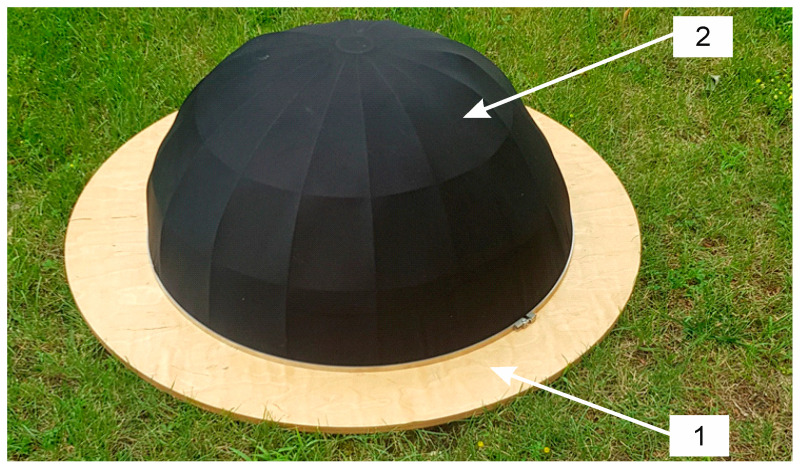
Image of external windscreen (2) installed on the reflecting board (1).

The second outer windscreen, which was also in the shape of a hemisphere with a radius of 45 cm, is part of the Brüel & Kjær UA-2133 probe set (Figure 5). The screen was made of an aluminum frame on which a nylon dome was placed. The cover was attached to a 1 m diameter round plate made of 12 mm thick waterproof plywood in order to separate the microphone from ground vibrations. In its central part, a microphone was placed in specially made plastic clamps, the measuring tip of which was directed towards the tested wind turbine. The use of the same reflecting surface during all measurements additionally minimized the effect of the soil surface on the recorded waveforms. Moreover, placing the microphone directly on the reflecting surface on the ground level additionally reduces the influence of gusts of wind on the recordings.

On the basis of the rated data regarding the equipment applied in the measurement system, the value of the type B standard uncertainty was estimated at 0.51. Moreover, the expanded uncertainty of B type was calculated and was equal to 0.92, with the assumed confidence interval of 95.5% [39].

## 4. Methodology of Measurements

**Figure 6:**
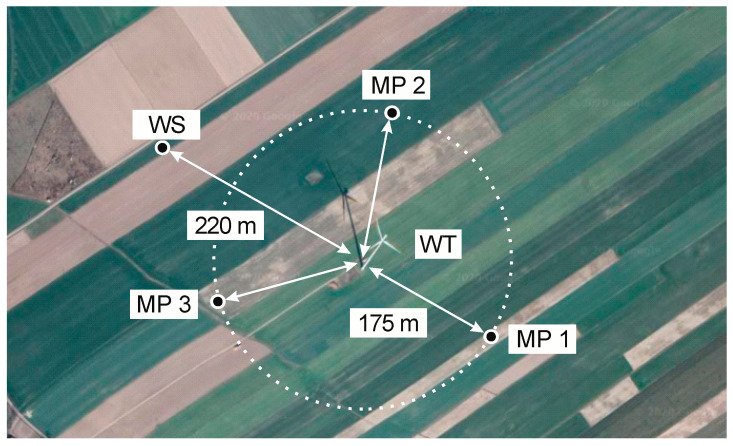
View of the location of the tested wind turbine and the distribution of measurement points, where: MP1-MP3—measurement points; WT—wind turbine under study and WS—weather station.

Figure 6 shows the location of three measuring setups in the field (named: MS_1; MS_2; MS_3), which were located at a distance of around 175 m in relation to the investigated wind turbine (denoted as: WIND_TURBINE). There were plowed farming fields around the turbine. There were no obstacles between the measuring stations and the analyzed turbine, and the area was virtually flat and devoid of vegetation.

**Figure 7:**
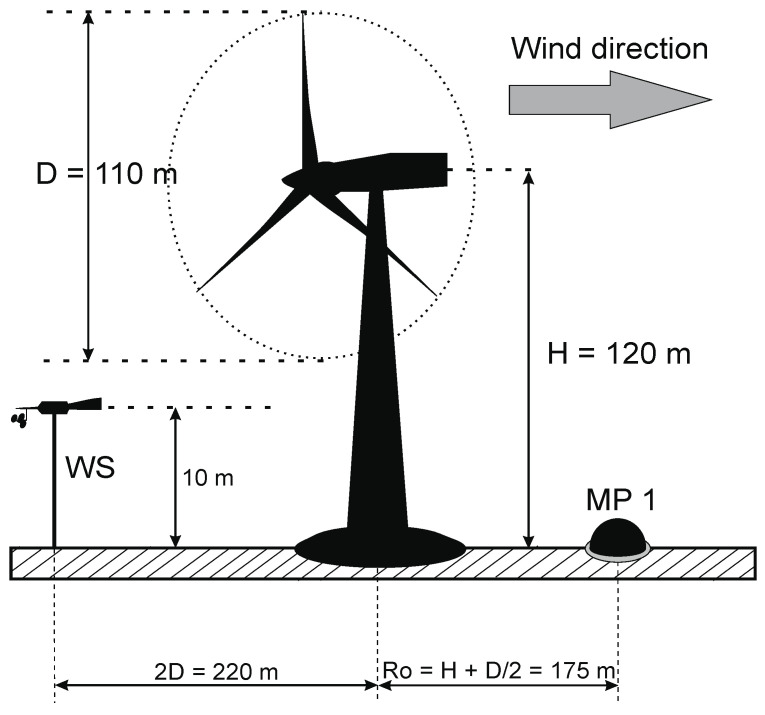
Location of the point of measurement (MP1) and WS in relation to investigated wind turbine on the basis of recommendations of IEC/EN 61400–11 standard, where: D—diameter of wind turbine rotor and H—height of turbine mast.

Figure 7 presents the layout of the measuring point (MP1) and the location of the weather station (WS) in relation to the tested wind turbine, which depends on the rotor diameter, D, and the height of the wind turbine tower, H, and results from the guidelines contained in the IEC/EN 61400–11 standard.

The infrasound measurements were carried out in early autumn, at the turn of September and October and took eight days. Signals were recorded in series taking total of 15 to 30 min each for every location of the measurement point so as to maintain a constant speed and steady wind direction. The measurements were performed during rainless weather and involved synchronized recording of infrasound signals from three measurement setups located in different locations in relation to the tested wind turbine. Apart from the simultaneous measurement of the emitted acoustic signals from three different measurement points, synchronized recordings of wind direction and speed were performed separately for each of the three measurement setups. The measured physical quantity involved the variations in the noise level, which were recorded simultaneously in three measurement setups. In this way, it was possible to determine the potential range of infrasound impact directly at the location of the measurement sites. The signals were recorded at a sampling rate of 51.2 kS/s. During the measurement days, the following values of the basic weather parameters were recorded: The mean value of the wind speed in the range from 3.4 to 12.3 m/s, atmospheric pressure in the range from 994.7 to 1001.2 hPa, air temperature ranged from 8 to 12 °C and its humidity was within in the range: 73–82%.

In accordance with the recommendations contained in the international ISO 389-7 standard and the recommendations contained in the IEC 61400-11 standard, prior to and after each measurement series, device calibration was performed for a given location and separately for each of the three measuring setups. For this purpose, the analysis applied a class 1 acoustic calibrator manufactured by B&K, type 4231. The calibration signal had a frequency of 1 kHz with a level of 94 dB, with its level stability being ±0.2 dB. On the other hand, the stability of the generated frequency, with distortions less than 1%, was equal to ±0.1%. The calibrator used meets the requirements of IEC 60942 class 1.

On each measurement day, background measurements were performed in the conditions of the stalled wind turbine. The values of reference signals obtained in this way were subtracted from the values of infrasound signals recorded during normal operation of the investigated wind turbine. The effect of the disturbances resulting from vehicular traffic, as well as by agricultural machinery operating in the fields and specialized vehicles used by foresters was eliminated by recording the time of their occurrence in order to remove them later from further analysis of the data recorded during measurements. Additionally, an occurrence of unusual variations in the frequency spectrum of the recorded infrasound and large fluctuations in their dynamics was recorded at a given stage of the analysis, the applied software made it possible to listen to a given section of the recorded signal, which in turn provided the possibility of identifying sources of disturbance and their effective elimination.

## 5. Results and Discussion

**Figure 8:**
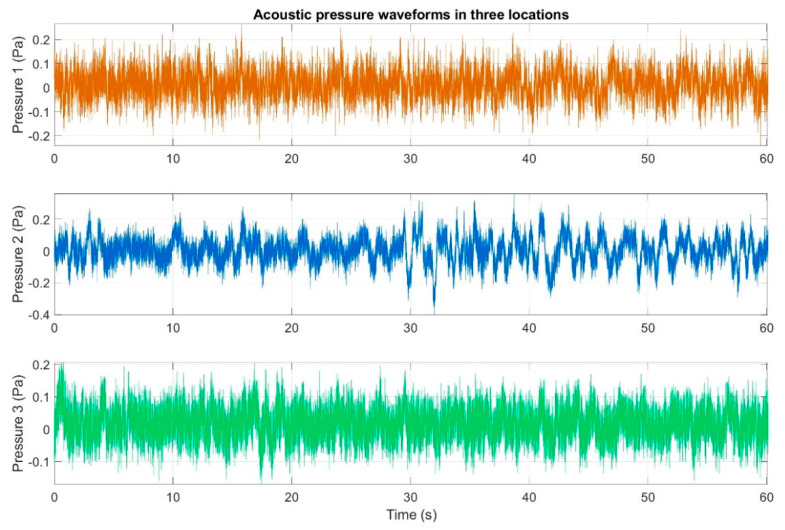
Acoustic pressure waveforms in three different locations measured simultaneously: Orange color—measuring setup number 1; blue color—measuring setup number 2 and green color—measuring setup number 3.

Figure 8 contains an example and representative waveforms of variations in the acoustic pressure occurring over one-minute intervals, which were recorded separated by the three measurement setups. One-minute time intervals were randomly selected from the measurement series with a duration of 15 to 30 min, during which the wind direction and speed were found to be constant during the performed registrations. At the same time, the dependencies presented in the article concern the data recorded for the wind speed of 12.3 m/s, which was the highest value for which case the infrasound signals measurements were performed. We should note that this is the value at which the tested turbine assumes its rated operating parameters. The wind direction was from the northeast.

**Figure 9:**
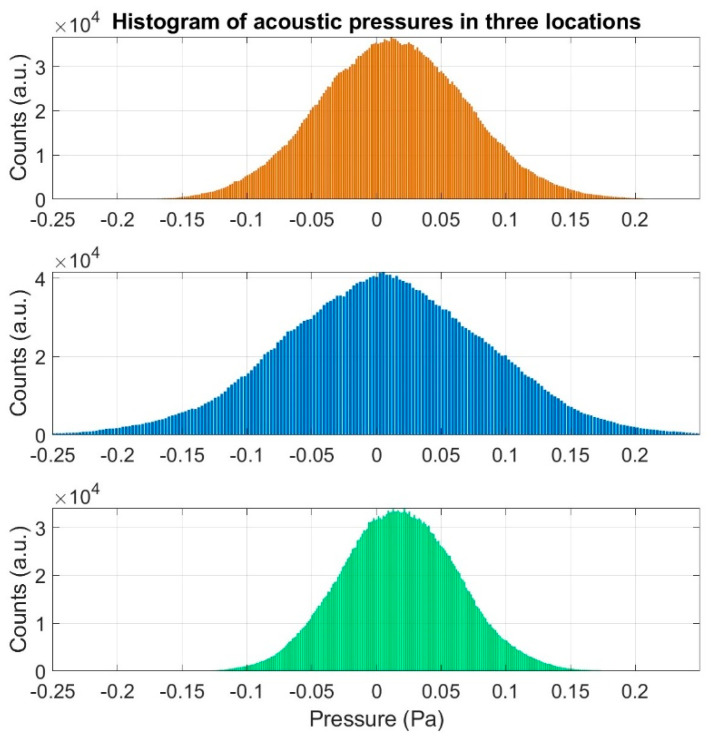
Histogram estimated for acoustic pressure acquired in three different locations at the same time: orange color—measuring setup number 1; blue color—measuring setup number 2 and green color—measuring setup number 3.

For the waveforms presented in Figure 8, histograms of acoustic pressures were determined separately for three locations. The obtained distributions are illustrated in Figure 9.

**Figure 10:**
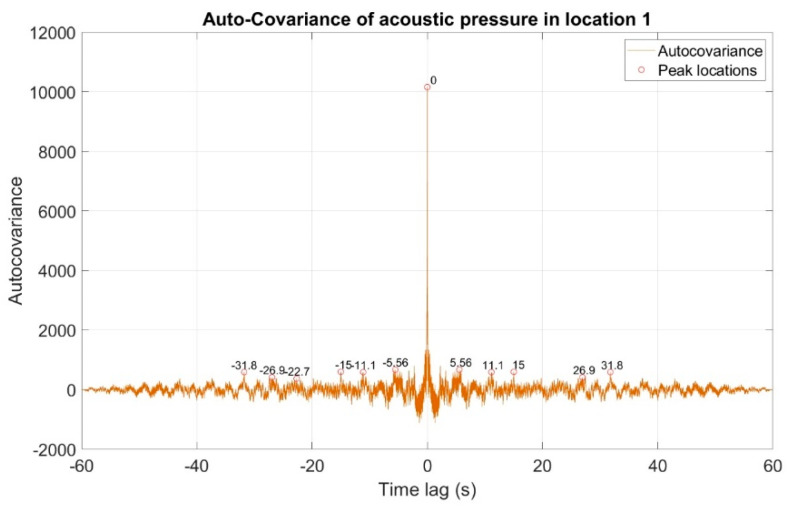
Course of auto-covariance function of infrasound signals generated by the investigated wind turbine, recorded in measuring setup number 1.

**Figure 11:**
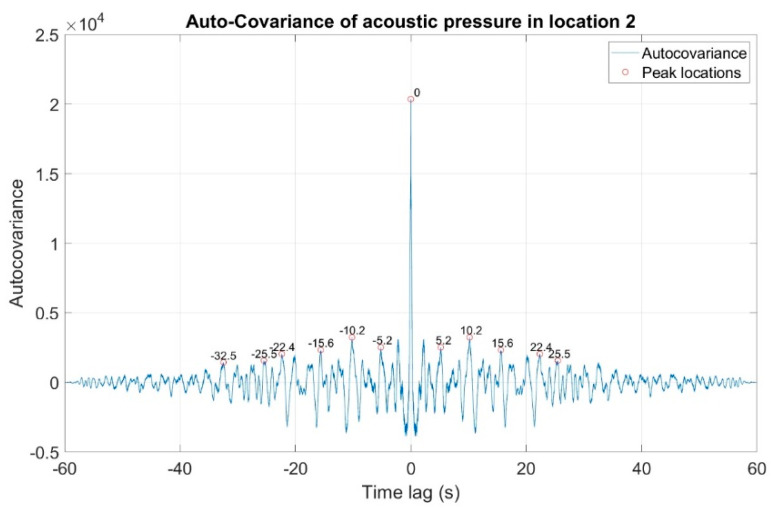
Course of auto-covariance function of infrasound signals generated by the investigated wind turbine, recorded in measuring setup number 2.

**Figure 12:**
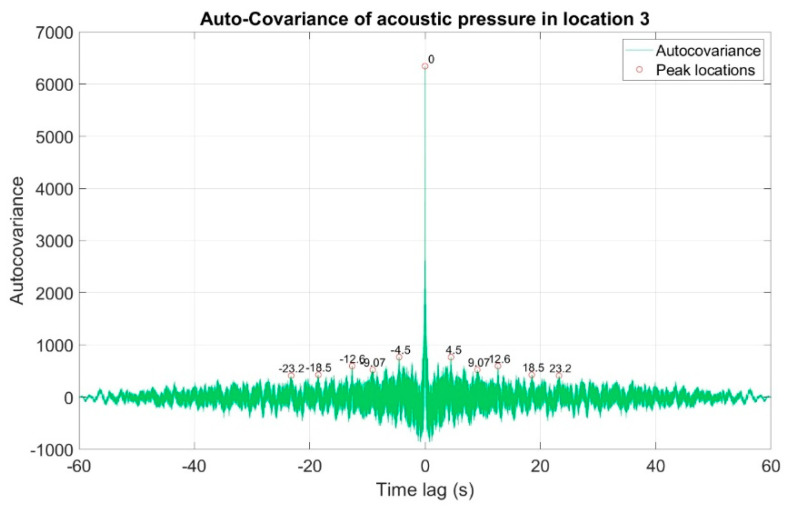
Course of auto-covariance function of infrasound signals generated by the investigated wind turbine, recorded in measuring setup number 3.

**Figure 13:**
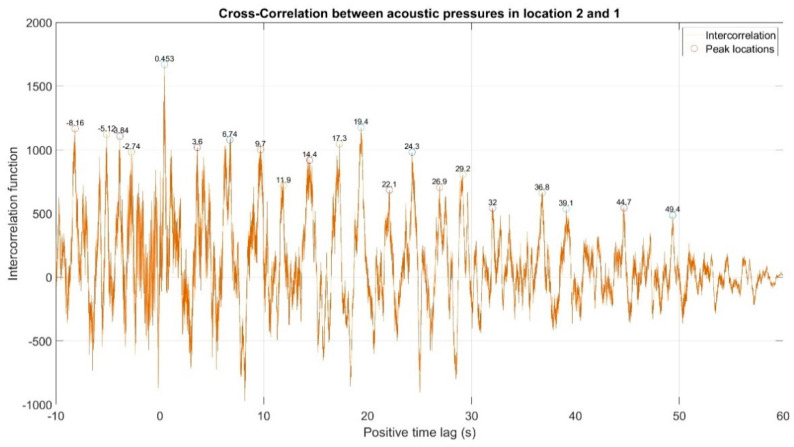
Inter-correlation between acoustic pressures in location 2 and 1. There are several peaks for positive lag. The highest correlation is for lag of 0.45 s.

**Figure 14:**
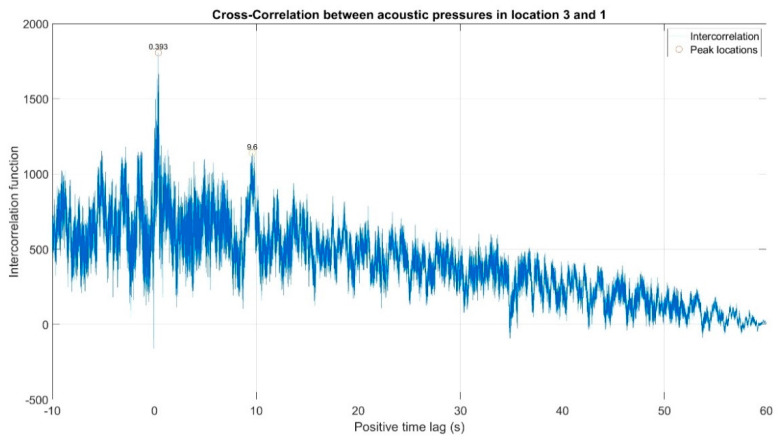
Inter-correlation between acoustic pressures in location 3 and 1. There are few peaks for positive lag. The highest correlation is for lag of 0.39 s.

**Figure 15:**
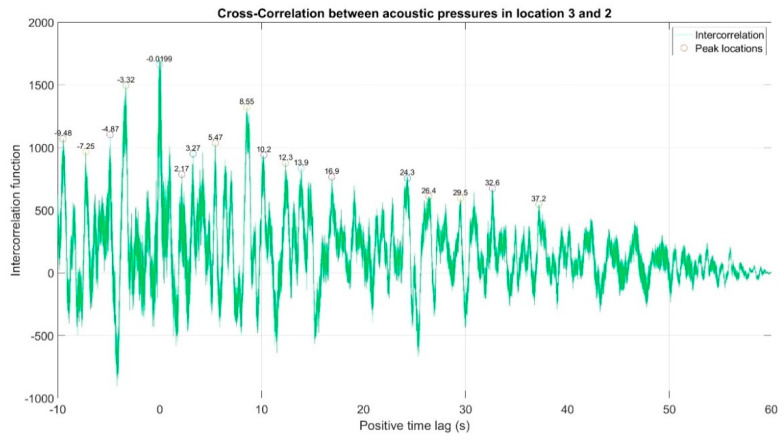
Inter-correlation between acoustic pressures in location 3 and 2. There are several peaks for positive lag. The highest correlation is for lag of −0.02 s.

The first stage of the analysis involved the recording of infrasound signals by the three measuring setups, whose waveforms are presented in Figure 8, followed by the subsequent correlation analysis in the time domain. For this purpose, the waveforms of the auto-variance function (auto-correlation with the subtracted mean value) were determined separately for each of the three measuring setups (Figure 10, Figure 11 and Figure 12). Moreover, the courses of the cross-correlation function were calculated separately for each of the possible combinations of infrasound recorded in the setups (Figure 13, Figure 14 and Figure 15). The use of correlation analysis in the time domain provides the possibility of identification of the characteristics of the recorded signals, and indicate the ratios of deterministic and stochastic components. Moreover, it can be used to identify the quantities accompanying the noise and interference measurements. Additionally, in order to determine the relations between the acoustic pressure changes for different time shifts, the cross-correlation functions were determined, taking into account subsequent possible combinations of two of the three measuring setups.

The second stage involved the analysis of the recorded infrasound signals in the frequency domain that was carried out by determining the waveforms of the power density spectra using the Welch method and Hamming window from the length N = 512e^3^. For this purpose, computational scripts that were developed in the Matlab programming environment. In detail, the methodology of calculations and the used mathematical relationships proposed by P.D. Welch for the estimation of power density spectra, e.g., in [40]. We should emphasize that this method was used in this article primarily to reduce the level of noise in the estimated power density spectra, which is the case when using the standard fast Fourier transform and to calculate the power density spectra or energy, respectively.

**Figure 16:**
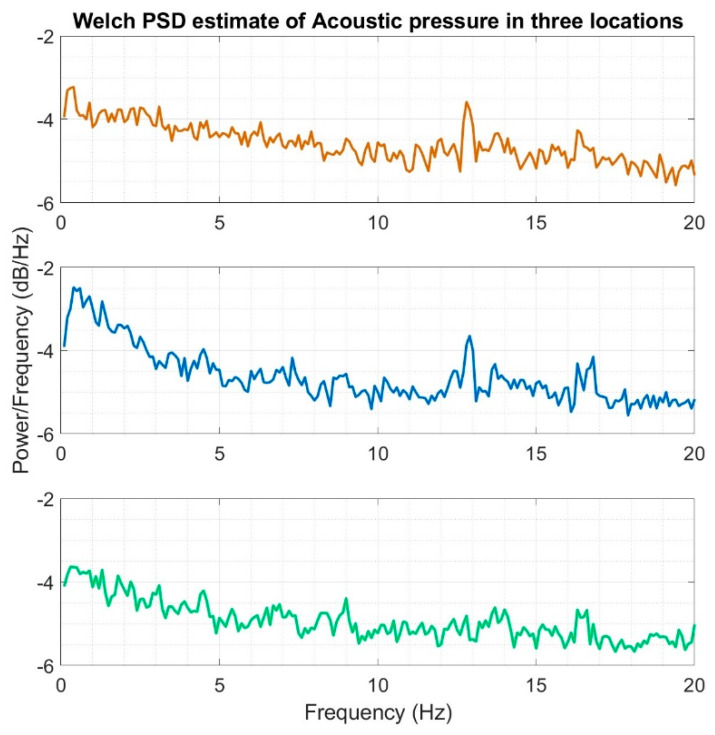
Welch power spectral densities estimated for acoustic pressure acquired in three different locations at the same time: orange color—measuring setup number 1; blue color—measuring setup number 2 and green color—measuring setup number 3.

We can also remark that the relations developed for lower wind speeds do not differ in terms of quality, as the characteristics of the determined waveforms of frequency spectra were maintained. However, lower amplitude values of the recorded infrasound signals were recorded. Figure 16 contains averaged waveforms of power density spectra, which were determined separately for each of the three setups.

**Figure 17:**
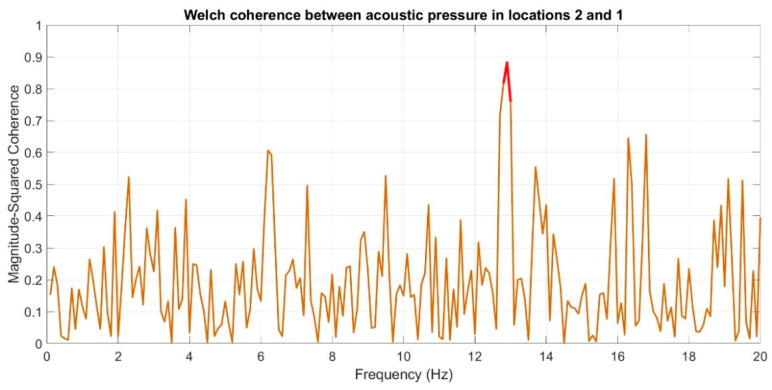
Coherence estimate via the Welch method for acoustic pressures in locations 2 and 1. The coherence above 0.75 is marked with red line.

**Figure 18:**
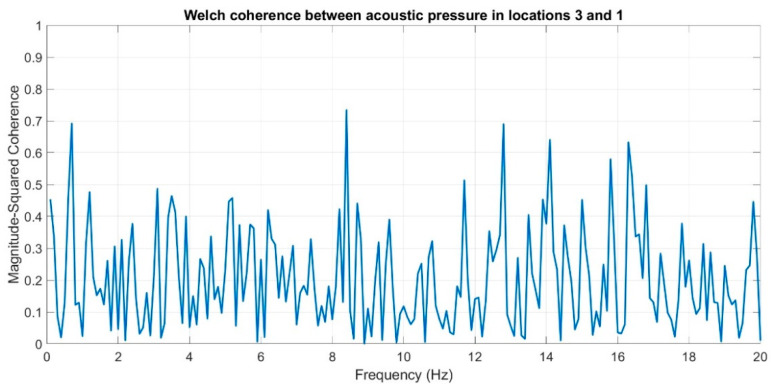
Coherence estimate via the Welch method for acoustic pressures in locations 3 and 1.

**Figure 19:**
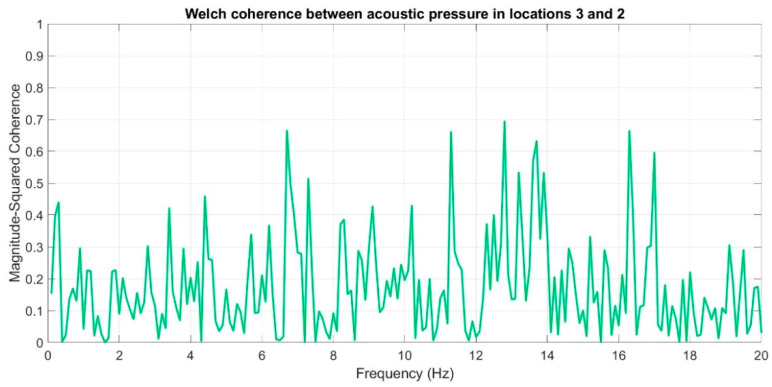
Coherence estimate via the Welch method for acoustic pressures in locations 3 and 2.

Subsequently, coherence function was used in order to determine the similarities in the frequency domain for the recorded infrasound signals, whose waveforms are presented in Figure 17, Figure 18 and Figure 19. The analysis was carried out by comparing the obtained results, pairing, successively, two measuring setups.

**Figure 20:**
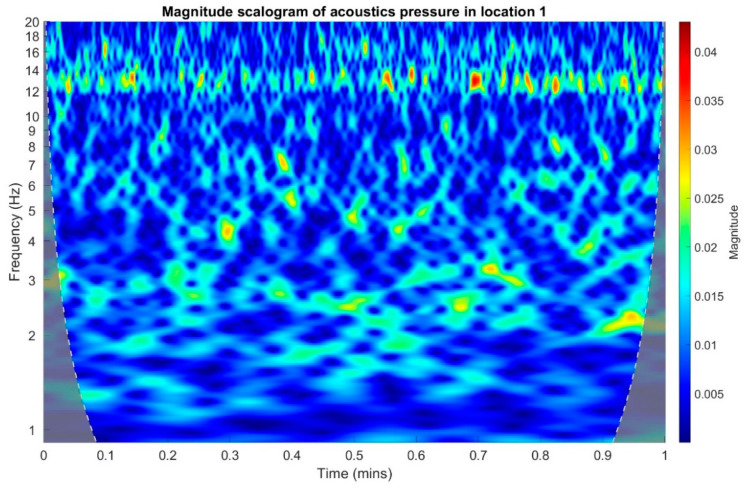
Magnitude scalogram of acoustic pressure in location 1.

**Figure 21:**
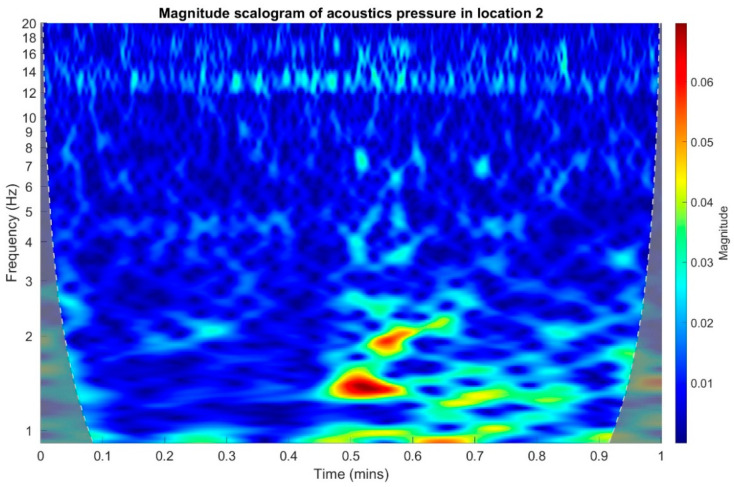
Magnitude scalogram of acoustic pressure in location 2.

**Figure 22:**
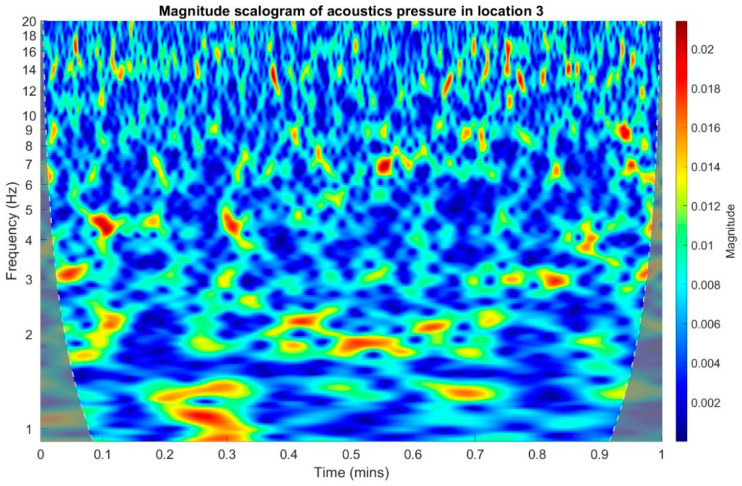
Magnitude scalogram of acoustic pressure in location 3.

In the following stage, infrasound signals recorded in three measuring setups were subjected to time-frequency transformations. The waveforms of the scalograms presented in Figure 20, Figure 21 and Figure 22 were determined using a continuous wavelet transform. Its use offers an increase the time-frequency bandwidth when the results are compared to the STFT (Short-time Fourier transform) transform, as it enables the use of narrow observation windows at high frequencies coupled with sufficiently wide for low frequencies. A Morlet wavelet was used as the base wavelet for determining the scalograms.

The courses of the coherence functions demonstrate that the highest correlation of frequency occurs between the courses recorded in setups number one and two. However, for setups three and two (Figure 19) and three and one (Figure 18), there is no coherence with a value above 0.75.

In an analogous manner to the time and frequency analysis of the recorded infrasound signals, wavelet coherence functions were determined to identify the correlations of the scalograms in the time-frequency domain. Wherein, the wavelet transform coherence (WTC) is a method for analyzing the coherence and phase lag between two time series as a function of both time and frequency. The wavelet coherence is define as [41]:

(1) $$R^{2}\left(\tau ,f\right)=\frac{{\left|S\left(\frac{1}{s}W_{xy}\left(\tau ,\;s\right)\right)\right|}^{2}}{S\left(\frac{1}{s}{\left|W_{x}\left(\tau ,\;s\right)\right|}^{2}\right)\cdot S\left(\frac{1}{s}{\left|W_{y}\left(\tau ,\;s\right)\right|}^{2}\right)}$$

(2) $$0\leq \;R^{2}\left(\tau ,f\right)\leq 1$$

where:

$W_{xy}$ is the cross-wavelet transform:

(3) $$W_{\mathrm{xy}}\left(\tau ,\;s\right)=W_{x}\left(\tau ,s\right)W_{y}^{*}\left(\tau ,s\right)$$

*W_x_* is the continuous wavelet transform:

(4) $$W_{x}\left(\tau ,s\right)=\overset{\infty }{\underset{-\infty }{\int }}x\left(t\right)\psi_{\tau ,s}^{*}\left(t\right)dt$$

where:

τ is the translation parameter and *s* is the scale parameter.
$\psi_{\tau ,s}^{*}$ is the complex conjugate function of mother wavelet scaled and translated by τ and *s*.
*s* is the smooth operator.

**Figure 23:**
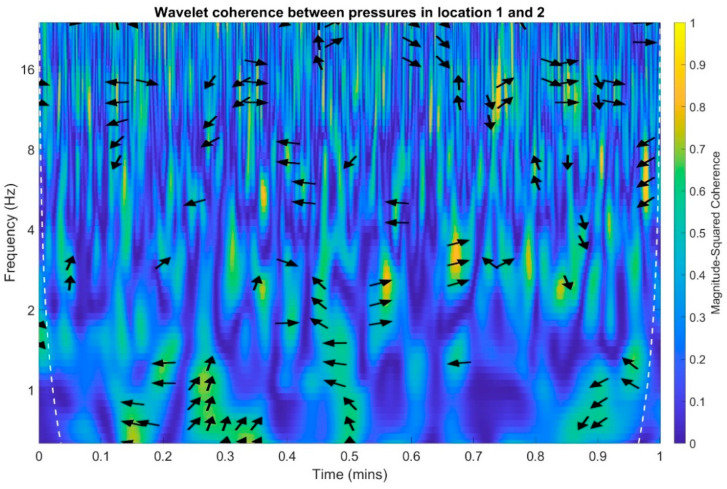
Wavelet coherence between acoustic pressures of infrasound measured in locations 1 and 2.

**Figure 24:**
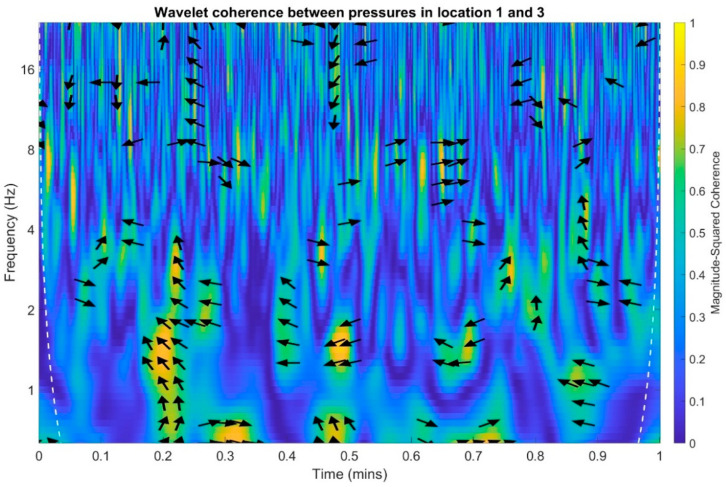
Wavelet coherence between acoustic pressures of infrasound measured in locations 1 and 3.

**Figure 25:**
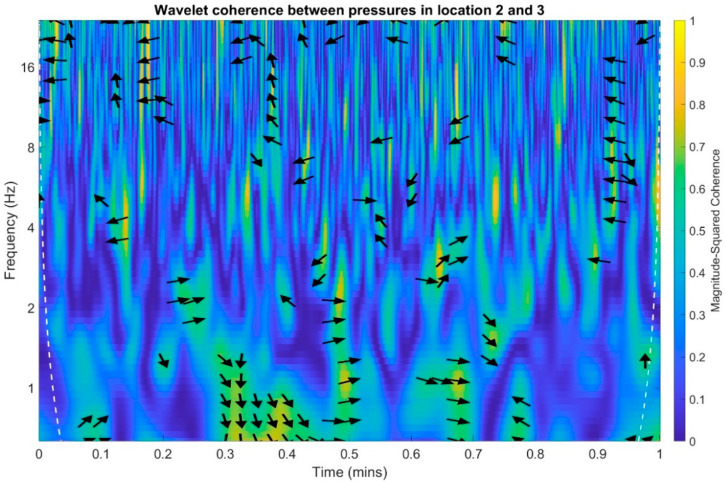
Wavelet coherence between acoustic pressures of infrasound measured in locations 2 and 3.

The wavelet coherence is a squared correlation localized in time and frequency. Calculations were performed separately for each of the three possible combinations of measuring setups, and the results are presented in Figure 23, Figure 24 and Figure 25. The arrows in the designated regions on the wavelets illustrate the variations in the correlation coefficient in time and frequency.

For examples arrow pointing to the right indicate that pressure in location one and pressure in the location two are positively correlated. Arrows pointing to the left indicate that pressures in the location one and two are negatively correlated. The straight up arrow implies that pressure in location one is leading in respect to location two. The straight down arrow imply that the pressure in location one is lagging in respect to pressure in location two (Figure 23).

On the basis of the conducted analysis, we can state the following conclusions:

- The waveforms containing variations in the acoustic pressure values presented in Figure 8 demonstrate the existence of relatively high levels of noise in the recorded infrasound signals, which has been confirmed by the designated histograms (Figure 9), where the shape is characteristic of Gaussian white noise.
- The calculated autocovariance waveforms (Figure 10, Figure 11 and Figure 12) are characterized by the presence of both a broadband stochastic component with a variable amplitude regardless of the setup applied for data recording. This component is visible in the entire range of the analyzed waveforms, and is combined with the presence of a deterministic component with a relatively high value visible for a zero shift. This is true of all three autocovariance courses. In addition, the numerical values of time shifts were marked on the designated waveforms, for which individual peaks were identified, the values of which differ from the noisy, time-blurred waveform. It should be noted that these values are definitely smaller than the value determined for the zero shift (at least several times).
- For the purposes of the easier identification of the common harmonics with the highest values that correspond to the greatest time correlation of the infrasound signals recorded in individual setups, the values of the corresponding time shifts have been marked on the cross-correlation functions presented in Figure 13, Figure 14 and Figure 15. In addition, several peaks for positive lag are visible in the determined course of the cross-correlation function between acoustic pressures in location two and one presented in Figure 13. The highest correlation was recorded for the lag of 0.45 s. In the case of inter-correlation between acoustic pressures in location three and one (Figure 14), a few peaks for positive lag were identified. The highest correlation occurs for the lag of 0.39 s. In contrast, for the course of inter-correlation function between acoustic pressures in location three and two (Figure 15). There are several peaks for positive lag. The highest correlation is for lag of −0.02 s. The presented waveforms demonstrate the occurrence of the greatest time correlation between the waveforms recorded in setups number one and two and three and two, respectively, and relatively smaller for setups number three and one.
- The variations in wind speed in the range from 3.4 to 12.3 m/s did not affect the waveforms of the recorded power density spectra (Figure 16). These issues were the subject of other studies described in the article [42], therefore they were not analyzed in detail in this case. The recorded range of wind speed applied practically the entire range of the capacity of the examined turbine, starting from its start-up at a starting speed of 4 m/s, until the rated parameters were reached for the speed of 12.0 m/s (measured at the height of the rotor hub). As a result of increasing the wind speed, an increase in the values of the determined amplitude spectra occurred as well, which did not exceed 12 dB. These relations are identical for the data recorded in each of the three measurement setups and are independent of the rotor orientation and the angle of the turbine blades inclination.
- The determined waveforms of power density spectra take a course that is similar to an inverted, asymmetrical parabola. From the frequency of 0.1 Hz, an increase in the values of the calculated spectra is visible, up to the extreme, which is achieved at a frequency close to 1 Hz. Subsequently, a systematic, almost exponential, decrease of this value occurs until the end assumed at about 10 Hz, above which the waveform is almost flat. In the range from 10 to 20 Hz, single resonance peaks are noticeable. They are in particular visible for the frequency of 13 Hz (for setups number 1 and 2) and for approximately 16.5 Hz (for all three setups).
- The dominant frequency bands in the range from 2 to 4 Hz are visible on the scalograms obtained on the basis of the study. There is also a noticeable increase in power for frequencies in the range of around (6–8) Hz and from about 12 to 16 Hz. We can also note that the highest values in the spectra occur for infrasound frequencies. Above 20 Hz, the power density value drops sharply by more than 6 dB.
- The coherence above 0.75 is marked with red line (Figure 17). There is an evidence of correlation in the range of 13 Hz. There is no evidence of coherence higher than 0.75 (Figure 18). There is no evidence of coherence bigger than 0.75 (Figure 19).
- In Figure 23 for times in the range from 0.6 to 0.8 exist several regions of positive correlation above 0.9 in the frequency range of 2–3 Hz and 12–16 Hz. In the example, there exists a region with negative correlation in the frequency range of 6–8 Hz. In general, there are more positive correlation over the negative correlation areas. On the Figure 24 we can see the negative correlation for the time of 0.2 s and frequency range 1 to 3 Hz. Furthermore, for time 0.5 s and frequency range 1–2 Hz. Moreover, there are several smaller areas of negative correlation mainly for frequency lower than 2 Hz and higher than 8 Hz. There are few areas of positive correlation mainly for frequencies in the range from 4 to 8 Hz e.g., for 0.7 s. In general, there are more negative correlation over the positive areas. In Figure 25 we can see straight down arrows for times 0.3–0.4 s which means the pressure in location two is lagging in respect to location three. There are several time-frequency areas of positive correlation. In general, positive correlation in for lower frequencies below 2 Hz. And negative correlation is for higher frequencies from 4 to 16 Hz. Between locations there can exist both positive and negative correlation. Usually, lower frequencies are more positively correlated and for higher frequencies the correlation is more negative for higher frequencies where changing the distance of pressure sensors. There are only few areas which would evidence the lead of lag pressure signal between locations.

## 6. Conclusions

As a result of the application of a wireless measuring system comprising three separate setups, it was possible to record the occurrence of variations in the low-frequency signals emitted by a wind turbine, simultaneously in any three directions, at the distances up to 300 m from the place of their generation. These types of measurements are not possible using professional measuring equipment that uses existing wired communication. We can note that the tests carried out explicitly confirmed its practical usefulness for measurements carried out during normal operation of wind installations, in often very demanding field conditions.

In the summary, we can state that the measurements performed by using only a single measuring setup, in a similar way as described in the recommendations specified in the standard [PN-EN 61400-11] applicable to the methodology of acoustic signals measurements, in particular in the audible bandwidth, does not take into account the possibility of propagation of infrasound waves in different directions in relation to the analyzed source. As a consequence, the resulting information about the studied phenomenon can be incomplete or even incorrect.

However, simultaneous measurements of infrasound signals performed in three different locations in relation to the investigated wind turbine makes it possible to obtain a mean of the values of measurements followed by option of verifying the results. In addition, it is possible to assess the influence on the obtained dependencies of such parameters as: the position of the turbine rotor axis in relation to the direction of the wind blow, other operating wind installations, terrain and other environmental parameters on the recorded infrasound signals, which is not possible by application of only one measuring setup. The research carried out by these authors aims to develop a model of the propagation of infrasound waves emitted by wind turbines on the basis of the measurement programs executed during normal operation of wind turbines. The use of correlation analysis in the time, frequency and time-frequency domains according to the methodology described in the article makes it possible to find the occurring similarities and, respectively, differences in the obtained relationships illustrated on time courses, frequency spectra and wavelet scalograms.

## Figures and Tables

**Table 1:** Summary of basic parameters of GRAS 46AZ microphone.

Parameter	Unit	Value
Frequency range (±1 dB)	Hz	1–10 k
Frequency range (±2 dB)	Hz	0.5–20 k
Sensitivity	dB(A)	17
Dynamic range lower limit with GRAS preamplifier	dB	138
Dynamic range upper limit with GRAS preamplifier	mV/Pa	50
Nominal sensitivity at 250 Hz	mA	2–20
Input current (CCP)	°C	od −30 do +70
